# Peripheral Nerve Regeneration: Opportunities and Challenges

**DOI:** 10.26502/fjsrs0052

**Published:** 2023-02-08

**Authors:** Rajiv Supra, Devendra K. Agrawal

**Affiliations:** 1 College of Osteopathic Medicine, Touro University, Henderson, Nevada; 2 Department of Translational Research, College of Osteopathic Medicine of the Pacific, Pomona, California

**Keywords:** Allografts, Autografts, Erythropoietin, Exosomes, Nerve conduits, Neuronal injury, Peripheral nerve injury, Peripheral nerve regeneration, Pharmacotherapy, Stem cells, Tacrolimus

## Abstract

Peripheral nerve injury has detrimental effects on the quality of life for patients and is a worldwide issue with high rates of morbidity. Research on the molecular mechanisms of nerve injury, microsurgical techniques, and advances in stem cell research have led to substantial progress in the field of translational neurophysiology. Current research on peripheral nerve regeneration aims to accelerate peripheral nerve development through pluripotent stem cells and potential use of smart exosomes, pharmacological agents, and bioengineering of nerve conduits. In this article critically reviewed and summarized various methods used for peripheral nerve regeneration and highlight the opportunities and challenges that come along with these strategies.

## Introduction

Peripheral nerve injury is a clinical problem with detrimental consequences that results in roughly 150 billion dollars in annual costs for injuries to the median and ulnar nerve; about 87% of these costs being from lost production [1]. Etiologies of peripheral nerve injuries include vibration injuries, ischemia, penetrating trauma, and electrocution [2]. In a civilian setting, motor vehicle accidents, lacerations by sharp instruments, or bone fractures are the most common mechanisms of injury in peripheral nerve damage [3]. Explosives and injuries from gunshots are the main reasons for peripheral nerve injury in a warfare setting [4]. Although patients are treated for peripheral nerve damage, many do not go on to full recovery resulting in loss of sensory and motor function [5].

Peripheral nerve surgery has been a major modality of treatment in recent years. Refinements in microsurgical techniques has improved clinical management in patients with peripheral nerve injury. A deeper understanding of the molecular basis of neuron growth has allowed for improved functional outcomes [6,7]. Direct end to end microsurgical epineural nerve repair has been the gold standard for treating peripheral nerve damage in settings with good blood supply and soft tissue [8]. Microsurgical repair requires clean-cut nerve transection with minimal neuron loss and mobilization of distal and proximal stumps [6]. When primary surgical intervention does not allow for repair of peripheral nerves, autogenous nerve grafting, artificial nerve conduits, pharmacologic agents, and stem cell therapies have shown potential in regenerating peripheral nerves [9]. This article aims to provide a critical overview of current research on peripheral nerve regeneration and future perspectives.

## Nerve Repair with Grafts

Significant gaps between nerves of greater than 2 cm makes primary end to end nerve repair obsolete. Such gaps present after considerable damage for example from gunshot wounds [4]. In cases of severe damage and enlarged gaps, nerve grafting has become the treatment of choice. This process involved harvesting a portion of a nerve and suturing it between proximal and distal stumps at the site of injury. Nerve stumps are first trimmed of excess scar tissue in which motor and sensory fascicles are then properly aligned [7]. The nerve graft is then placed between the gap with some redundancy in the repair in case the nerve is in proximity with flexion of a joint. Autografts and allografts are the methods used for nerve grafting [6]. Schwann cells (SCs) are provided by autografts and are essential for axon regeneration. Factors such as size of nerve gap, location of nerve repair, and donor-site morbidity are considered and can affect the choice of autogenous nerve grafting [10,11]. Grafts are sutured to the epineurium of nerves depending on the nerve type and location. Nerve autografts are harvested from sensory nerve sites that are expendable. The sural nerve, for example, is a major graft used for peripheral nerve repair. It enables roughly 40 cm of graft to be taken from each leg. Alternative sites include lateral and medial cutaneous nerves of the forearm, superficial and deep peroneal nerves, posterior and lateral cutaneous nerves from the thigh, and dorsal cutaneous branch of the ulnar nerve [12]. For upper extremity reconstruction, Ray et al. suggest using the anterior branch of the medial antebrachial cutaneous nerve [13]. Using nerve autografts involves sacrificing a functioning nerve that is otherwise expendable [14]. The nerve that is harvested for autografting undergoes Wallerian degeneration and provides mechanical structure for axons to grow [15]. Additionally, within the repair site, there are unavoidable issues such as fascicle mismatch, fibrosis of sutures, and damage to the nerve from tissue handling. All these factors in combination lead to poorer outcomes for patients [16].

Nerve allografts are also used as an alternative but require immunosuppression. Techniques used to reduce allograft antigenicity include lyophilization, irradiation, and cold preservation [17]. At approximately 24 months after nerve repair, migration of SCs occurs into the nerve allograft at which point systemic use of immunosuppressive agents can be withdrawn [6]. Nerve allografts go through a process of decellularization, a technique that degrades enzymes, resulting in a nerve scaffold that is acellular. The advantage of this technique compared with nerve conduits in the internal structure maintains an environment ideal for axonal regeneration and repair [9]. Recent research currently supports the use of decellularized nerve allografts for gaps up to 30 mm in length [18].

## Pharmacotherapy

Currently, no pharmacological agents have been used in a clinical setting, however many recent studies suggest pharmacotherapy as a potential strategy to accelerate nerve regeneration [19]. Potential pharmacologic agents for peripheral nerve regeneration are summarized in Table 1.

Erythropoietin (EPO), a cytokine that controls red blood cell production is often used in the settings of renal failure [20]. EPO, in the nervous system, has been investigated to prevent neuron apoptosis [21]. The mechanism of this, however, is yet to be understood. EPO is thought to be produced by perineuronal SCs in the peripheral nervous system and from increased expression of calcitonin gene-related peptide (CGRP), PI3K/Akt, JAK/STAT-3, and NF-kB signaling pathways get activated which promotes neuron survival [22]. Since EPO levels have been shown to decline after injury, EPO may be insufficient to provide continual neuroprotection. Studies reveal, however, that even after a week of post sciatic nerve injury, EPO promoted motor function improvement in murine models of peripheral nerve injury [23]. This reinforces the theory that prolonged administration of EPO may be required for peripheral nerve healing [24]. Additionally, EPO has been shown to have anti-apoptotic and anti-inflammatory effects in murine models of several conditions in the central nervous system. Its utility is being tested in phase 2 and phase 3 clinical trials for traumatic brain injury and spinal cord injuries [25].

Tacrolimus (FK506) is another promising pharmacological agent that can be used for peripheral nerve injuries. Tacrolimus is an immunosuppressant that acts through the inhibition of calcineurin [26]. It is currently being used for organ rejection treatments and has effects on nerve regeneration through the binding of FK506 to heat shock protein 90 (Hsp90) and protein 52 (FKBP-52) and activating the ERK pathway [27]. In murine models with nerve transection, short term administration of FK506 resulted in accelerated rates of nerve regeneration and improved functionality [28]. In rats with delayed nerve repair, FK506 reversed the effects of chronic axotomy on nerve regeneration [29]. Although FK506 has been shown to increase Wallerian degeneration through increasing the proliferation of SCs, the long-term effects are not beneficial because of extensive atrophy in SCs [30]. Due to the systemic side effects and immunosuppression due to FK506, it is difficult to justify its use in peripheral nerve repair. However, when FK506 was administered in a vein graft across a sciatic nerve gap, motor functionality increased as well as axon outgrowth [31]. In future studies, dose response studies need to be considered to minimize immunosuppression and maximize axon regeneration.

N-acetyl cysteine (NAC) is known for its antidote effects against acetaminophen toxicity as well as mucolytic effects [32]. Its mechanism of action includes donating cysteine in the synthesis of glutathione, and it can prevent apoptotic signaling in neuronal cells through the RAS-ERK pathway [33]. Bcl-2 is upregulated by NAC and the mRNA of pro-apoptotic proteins, such as caspase-3 and Bax, are downregulated [34]. In a murine model of sciatic and sural nerve transections, NAC administration resulted in increased survival of neurons [35]. Motor neurons have also been shown to be protected by NAC following rhizotomy and ventral root avulsion [36]. NAC also provided optimal nerve protection against cadmium-induced neurotoxicity [37]. Motor neurons following NAC administration, however, do not seem to respond similarly as peripheral nerves. Following sciatic nerve transection in murine models, no growth promoting effects was noticed on motor neurons in their growth into the distal nerve stump [38]. Since NAC is already used clinically, its clinical use for peripheral nerve regeneration is feasible. The neuroprotective effects NAC on humans is worth noting and the inability to promote axon regeneration in motor neurons is a subject that is yet to be determined through further research in experimental and clinically relevant animal models.

Geldanamycin, an antibiotic that was used as a chemotherapeutic agent, has also shown promising data as a neuroprotective agent. Geldanamycin binds strongly to Hsp90 similarly to tacrolimus [39]. In a murine saphenous nerve crush model, Geldanamycin was shown to accelerate the rate of axon regeneration and promoted earlier functional recovery in motor neurons after tibial nerve crush injury [40]. However, the poor water solubility and hepatotoxic side effects of Geldanamycin have made it difficult to use in human subjects [41]. Newer analogues of the drug are yet to be investigated.

## Stem Cell Therapy

The effect of stem cell therapy with or without in vitro incorporation into a biomaterial-based scaffold has been well investigated by many researchers. The tissue and nerve graft are sutured to the site of injury to bridge the nerve defect with local administration of stem cells. Following nerve graft transplant, stem cells differentiate into SCs and promote axon growth. Many different sources for stem cells have been applied and studied in nerve tissue engineering. For example, embryonic stem cells can differentiate into all embryonic germ layers and create all types of tissues in the body except for fetal cells. Neurospheres from human embryonic stem cells can differentiate into cells with similar features as SCs [42]. Embryonic stem cells that differentiate into SCs express SC markers, S100 and p75, and induce myelination of neurons in the dorsal root ganglion [43]. Microinjection of murine embryonic stem cell-derived neural progenitor cells into epineurium of transected sciatic nerves in rates led to substantial functional and morphological recovery. Injected stem cells can differentiate into SC-like cells months after they are transplanted [44]. Although embryonic stem cells have shown promising results in murine models, they have been shown to induce formation of teratomas [45,46]. Moreover, the use of embryonic stem cells has been known to trigger ethical controversy.

Mesenchymal stem cells are multipotent and can be found in many tissues of the body. Specifically, bone marrow mesenchymal stem cells (BMMSCs) can be retrieved from bone marrow aspiration and can be cultured. Cultured BMMSCs lack immune recognition and be transplanted to hosts without rejection, thus making it one of the most widely used sources for nerve repair [47,48]. BMMSCs can differentiate into SC-like cells, increasing neurite outgrowth [49]. Studies show when seeding BMMSCs into silk fibroin-based nerve conduits, the levels of S100, a SC marker, increased and subsequently elevated many growth factors which ultimately accelerated the recovery in sciatic nerve injury murine models [50]. Compared to the plain nerve graft, the acellular graft with BMMSCs exhibited faster rates of axon growth, and walking track when a 10 mm sciatic nerve defect was bridged in murine models [50]. Bone marrow stem cells have also been shown to increase angiogenesis through elevating levels of vascular endothelial growth factor [51]. Moreover, BMMSCs secrete neurotrophic factors like extracellular matrix proteins, collagen, fibronectin, and glial cell line-derived neurotrophic factor. When a silicone tube containing BMMSCs was transplanted in murine sciatic nerve gap models, rat walking behavior improved, muscle atrophy reduced, and axon regeneration was stimulated [52]. BMMSCs have also been shown to treat peripheral nerve injuries in primate. SCs induced by BMMSCs was filled into a nerve conduit with a collagen sponge to bridge the 20 mm median nerve injury in cynomolgus primates. When cells were transplanted, the growth of axons were observed without massive cell proliferation [53]. Additionally, another study revealed a nerve graft engineered with chitosan and BMMSCs resulted in better repair to a 50 mm median nerve injury than plain nerve conduits in primate models [54].

Studies using adipose stem cells (ASCs) have also shown promising data in nerve regeneration, although use has been limited to its ethical controversy. ASCs can be retrieved through less invasive methods and can exhibit greater clinical potential [55]. ASCs can differentiate into spindle shaped cells that can express SC markers and can stimulate the formation of myelin sheaths [56,57]. Biochemical studies using ASCs showed differentiation into SC phenotypes and improvement of mean amplitudes of muscle action potential and axon diameter after transplantation to bridge a 1 cm murine sciatic nerve gap. Fibrin conduits seeded with ASCs seemed to have better regenerative effects than primary SC-seeded fibrin conduits [58,59]. ASCs were also incorporated into silicone nerve conduits with a type I collagen gel to bridge a 7 mm defect of murine facial nerve defects. Results revealed increased myelinated fibers, myelin thickness, and an over functional improvement in the facial palsy [55]. Of note is the donor age and harvest site of ASCs that can greatly limit the growth properties of ASCs [60,61]. To maximize the regenerative potential of ASCs, the quality of ASC must be considered.

Although the stem cell therapy could have beneficial effects on peripheral nerve regeneration, number of potential drawbacks might limit their use. These drawbacks include low rate of cell survival and graft cell death, immune-mediated rejection, attenuated regenerative capacity of engrafted cells, tumorigenesis, and ethical and regulatory issues [62]. In recent years, the use of stem cell-derived or Schwann cell-derived exosomes, a subclass of extracellular vesicles, have shown some promise to mediate intercellular communication in tissue systems [63–65]. Cell-to-cell contact, and secreted signals (secretome) are critical in axon-glia interaction. Indeed, exosomes carry several constitutive molecules and cargo molecules, including proteins such as growth factors, mediators of gene expression including the transcription factors and cytokines capable of eliciting paracrine biological responses, lipids, and various genetic materials such as mRNA, miRNA, and traces of DNA [63–67]. However, many of the cargo materials in exosome could have off target effects. Therefore, it is critical to define an ideal cell type to derive exosomes and prepare “smart exosomes” by packing them with nerve regenerative mediators to increase myelin-related and neurogenic genes, enhance neurite outgrowth, induce migration and proliferation of Schwann cells, and decrease proinflammatory cytokines [68]. Additionally, the smart exosomes could be delivered using an effective delivery system such as minimally invasive intelligent hydrogels in peripheral nerve regeneration [69]. However, well designed, and controlled studies using these novel strategies are limited and warrant further investigation.

## Artificial Nerve Conduits

Although axon regeneration has been reported in gaps less than 5 mm, the functional recovery is poor without proper intervention [70]. In case of gaps less than 1 cm between the nerve injury, direct end to end suturing without tension will allow for proper regeneration. However, for defects greater than 3 cm, bringing the nerve ends together without creating substantial amount of tension is impossible. To overcome this limitation, nerve tissue transplant has been researched to region nerve segments [71]. The disadvantage of nerve grafts includes increased surgical complications, diameter mismatch between nerve and graft, and immunological reactions [72]. A nerve conduit has provided an alternative strategy to bridge the defective nerves and promote regeneration (Table 2).

Nerve conduits have been designed using nondegradable synthetic materials providing a safer option than donor grafts and eliminating the need of any immunosuppressant therapy [73]. Following implant of the surgical conduit, axons can regenerate from the proximal to distal stump [74]. Nerve conduits are designed to enhance peripheral nerve reconstruction and axon guidance as well as preventing formation of fibrous scar tissues [73,75]. Ideal nerve conduits have low immunogenicity, are bio-absorbable, and have substantial mechanical properties to maintain the alignment between proximal and distal nerve sites [76–78]. Different biomaterials have been studied and have been classified as natural polymers and synthetic materials [77,79].

Polyglycolic acid (PGA) is an FDA approved synthetic polymer used for nerve conduits. Using PGA allowed for more flexible and porous material which enhanced the nerve regeneration process [70,80]. The use of PGA nerve conduits allowed for better reconstruction of long nerve gaps compared to other synthetic polymer materials. Additionally, gaps less than 4 mm led to greater repair outcomes compared to end-to-end regeneration [81,82]. Polylactic acid (PLA) enhances neuron regeneration and is made from biodegradable polyester. PLA nerve conduits can support neuron proliferation and has been shown to improve maturation of axons [70]. PLA conduits, however, generate lactic acid as a byproduct during its degradation which can result in neurotoxicity. Further studies are needed to elucidate its efficacy in peripheral nerve regeneration [83,84].

Poly(L-lactide-co-ε-caprolactone) (PLCL) contains lactic acid. A transparent PLCL conduit can theoretically produce less lactic acid during degradation rendering it a more viable option considering its reduced toxicity. The efficacy of this conduit has been assessed with mixed outcomes. Studies show no promising effects of PLCL when promoting nerve repair in digital nerves, however PLCL has had comparable results in end-to-end repair. These conflicting findings potentially can be explained through the location of the nerve injury. The efficacy of PLCL remains in controversy and further studies are needed on this nerve conduit [85–87].

In addition to synthetic nerve conduits, natural polymers have also been used providing cell growth and support. Collagen and chitosan have been extensively used in nerve conduit fabrication and have minimal biodegradability and immunogenicity. Type I collagen has been the most widely used material and constructs fibrils of the endoneurium in the basal lamina. Collagen conduits have the ability to bridge nerve gaps up to 20 mm and improve functional outcomes in nerve reconstruction. Studies have also revealed reduced symptoms of pain [88–90]. Chitosan, another natural polymer, has also been researched for its ability to increase neurite growth and nerve cell adhesion [91–93]. Gu et al. revealed chitosan fibers promoting migration, adhesion, and proliferation of SCs and axon growth. A case study revealed chitosan repairing a 30 mm defect of the median nerve which resulted in restoration of motor function of thumb abduction [94]. Additionally, in murine models of nerve repair, chitosan has been shown to induce nerve repair in 1 cm gaps between nerves [94].

## Conclusion

This review summarized the current research on peripheral nerve repair to highlight current and developing perspectives in the growing field of nerve regeneration. As discussed, there are many opportunities in the use of potential pharmacologic agents for peripheral nerve regeneration, stem cell therapy, and artificial nerve conduits. Potential use of “smart exosomes” with minimally invasive intelligent hydrogels is promising in the regeneration of peripheral nerve. However, these strategies do not come without challenges and warrant careful investigation. Together with a greater understanding of the biology underlying nerve regeneration coupled with latest advances in biotechnology can result into promising breakthroughs in the field of nerve regeneration.

## Figures and Tables

**Table 1:** Potential pharmacologic agents for peripheral nerve regeneration

Pharmacologic Agent	Mechanism of Action	Effect	Reference
Erythropoietin	-Activates NFkB, JAK/STAT, and PI3K/Akt pathways	-Increases rate of axon regeneration	20,21,22
	-Increases calcitonin gene-related peptide	-Increases axon sprouting Neuroprotection	
		-Increases axon density and improves motor axon growth	
Tacrolimus	-Binds Hsp-90	-Increases myelinated axons	26,27,28
	-Calcineurin Inhibition	-Neuroprotection	
	-Induces proliferation of Schwann cells	-Increases axon regeneration	
N-acetylcysteine	-Decreases apoptotic signaling	-Reduces rates of neuronal death	32,33,35
	-Upregulates Bcl-2 mRNA	-Increases sensory nerve regeneration	
	-Downregulates Bax and caspase 3		
Geldanamycin	-Binds Hsp-90	-Increases rate of axon regeneration	39,40,41

**Table 2:** Characteristics of materials for nerve conduits and their applications

Material	Type	Characteristics	Application	Reference
Polyglycolic Acid	Synthetic	-Stable	-Nerve conduits	71,81,82
		-Biodegradable	-Nerve allografts	
Polylactic Acid	Synthetic	-Biocompatible	-Nanoparticles	83,84
		-Easy to fabricate	-Nerve conduits	
PLCL	Synthetic	-Low immunogenicity	-Nerve conduits	85,86,87
Chitosan	Natural	-Weak Degradability	-Nerve conduits	91,92,93
Collagen	Natural	-Degradable	-Nerve conduits	88,89,90
		-Biocompatible	-Membrane	

## Data Availability

Not applicable since the information is gathered from published articles.
